# Impact of party balloon inflation manoeuvre during saline contrast transthoracic echocardiography for patent foramen ovale detection: INFLATE-PFO registry

**DOI:** 10.1093/ehjimp/qyaf080

**Published:** 2025-06-12

**Authors:** Akihisa Kataoka, Masaki Izumo, Kento Kito, Taiga Katayama, Masataka Arakawa, Masashi Amano, Yasuhide Mochizuki, Hiroyuki Iwano, Yoko Nakaoka, Eiji Yamashita, Kiyoko Uno-Eder, Hirotsugu Yamada, Ken Kozuma

**Affiliations:** Division of Cardiology, Department of Internal Medicine, Teikyo University, 2-11-1 Kaga, Itabashi-ku, Tokyo 173-8605, Japan; Department of Cardiology, St. Marianna University School of Medicine, 2-16-1 Sugao, Miyamae-ku, Kawasaki, Kanagawa 216-8511, Japan; Division of Cardiology, Department of Internal Medicine, Teikyo University, 2-11-1 Kaga, Itabashi-ku, Tokyo 173-8605, Japan; Division of Cardiology, Department of Internal Medicine, Teikyo University, 2-11-1 Kaga, Itabashi-ku, Tokyo 173-8605, Japan; Department of Cardiology, Asahi General Hospital, I-1326, Asahi-shi, Chiba 289-2511, Japan; Department of Heart Failure and Transplantation, National Cerebral and Cardiovascular Center, 6-1 Kishibe-Shimmachi, Suita, Osaka 564-8565, Japan; Division of Cardiology, Department of Medicine, Showa Medical University School of Medicine, 1-5-8 Hatanodai, Shinagawa-ku, Tokyo 142-8666, Japan; Division of Cardiology, Teine Keijinkai Hospital, 1-12-1-40 Maeda, Teine-ku, Sapporo, Hokkaido 006-8555, Japan; Department of Cardiology, Chikamori Hospital, 1-1-16, Okawasuji, Kochi-shi, Kochi 780-8522, Japan; Department of Cardiology, Gunma Prefectural Cardiovascular Center, 3-12 Kameizumimachi-kou, Maebashi, Gunma 371-0004, Japan; Teikyo Academic Research Centre, Teikyo University, 2-11-1 Kaga, Itabashi-ku, Tokyo 173-8605, Japan; Department of Community Medicine for Cardiology, Tokushima University Graduate School of Biomedical Sciences, 2-50-1 Kuramoto-cho, Tokushima-shi, Tokushima 770-0042, Japan; Division of Cardiology, Department of Internal Medicine, Teikyo University, 2-11-1 Kaga, Itabashi-ku, Tokyo 173-8605, Japan

**Keywords:** abdominal compression, agitated saline contrast transthoracic echocardiography, party balloon inflation manoeuvre, patent foramen ovale, valsalva manoeuvre

## Abstract

**Aims:**

Paradoxical embolism from a patent foramen ovale (PFO) can cause cryptogenic stroke. Agitated saline contrast transthoracic echocardiography (ASC-TTE), with the Valsalva manoeuvre (VM), is crucial for diagnosing PFO. However, the VM is associated with false-negative outcomes. The party balloon inflation manoeuvre (PBIM) provides improved visualisation of provocation; however, its real-world efficacy remains uncertain. This study aimed to demonstrate the superiority of the PBIM over standard provocative methods (conventional or abdominal compression VM) during ASC-TTE.

**Methods and results:**

This multicentre retrospective observational registry study involved 117 consecutive patients (62.3% male; mean age, 56.2 ± 13.8 years) with PFO detected by transoesophageal echocardiography. During ASC-TTE, patients underwent PBIM and standard provocative methods. The primary endpoint was a five-point microbubble grading [representing a right-to-left (RL) shunt] change. Diagnostic performance of a significant RL shunt defined as Grade ≥2 in the five-point microbubble grading was assessed using McNemar’s test. Compared with standard provocative methods using Wilcoxon paired analysis, the PBIM resulted in microbubble upgrading in 51 patients, no change in 54, and downgrading in 12. The PBIM had a significantly higher microbubble grade than standard provocative methods [median, 3.0; interquartile range (IQR), 3.0–4.0 vs. 3.0; IQR, 1.7–4.0; *P* < 0.001]. For significant RL shunts, where transcatheter PFO closure would be clinically advised, PBIM improved diagnostic performance from 75.2% to 91.5% (*P* < 0.001).

**Conclusion:**

PBIM indicated transcatheter PFO closure in patients with cryptogenic stroke and suspected PFOs, particularly those with false-negative diagnoses for shunts. PBIM demonstrates greater efficacy than standard provocative methods during ASC-TTE, providing clinical practicality.

**Clinical Trial Registration:**

uMIN Clinical Trials Registry UMIN000051954 (https://center6.umin.ac.jp/cgi-open-bin/ctr/ctr_view.cgi?recptno=R000058907).

## Graphical Abstract

**Graphical Abstract:**
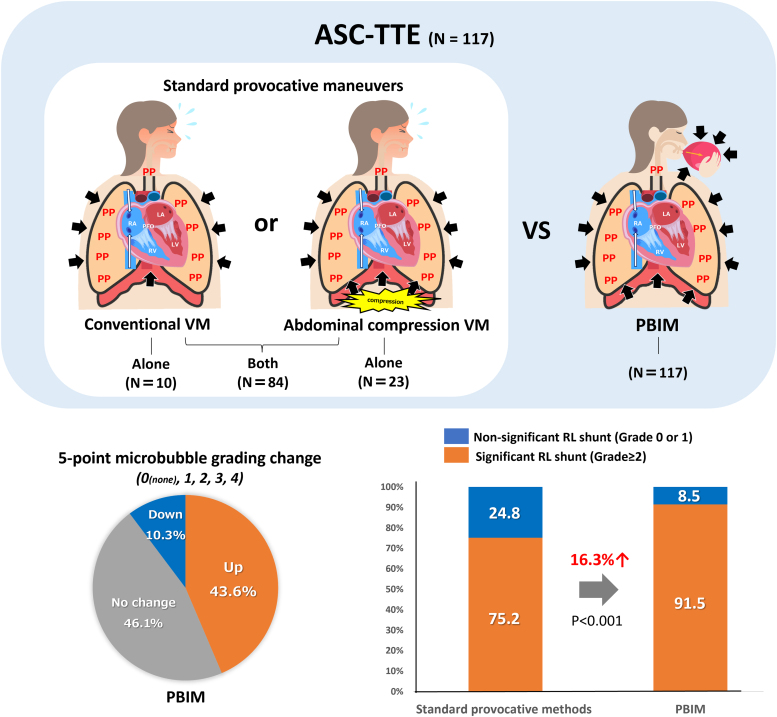

## Introduction

Patent foramen ovale (PFO) is associated with various diseases, including cryptogenic stroke (paradoxical embolism), migraine, and platypnea–orthodeoxia syndrome.^1–3^ Transcatheter PFO closure is used to manage PFO, and it significantly reduces stroke recurrence compared with medical therapy.^4–7^ However, identifying significant right-to-left (RL) shunts before transcatheter PFO closure is essential because their presence in PFO can vary dynamically. Agitated saline contrast transthoracic echocardiography (ASC-TTE) is a non-invasive screening tool that can detect a substantial RL shunt linked to cryptogenic stroke. ASC-TTE has greater sensitivity and precision than transoesophageal echocardiography (TEE), which causes insufficient Valsalva manoeuvre (VM) due to probe insertion.^8–11^ VM is the easiest and least invasive method. It is widely used in ASC-TTE as a standard provocative manoeuvre because it can induce haemodynamic changes in cases where an increased venous return to the right atrium (RA) and elevated RA pressure result in enhanced RL shunting through the PFO.^8^ The abdominal (inferior vena cava) compression VM has high sensitivity in detecting PFO and is widely performed in echo laboratories.^12,13^ However, a major challenge with these standard provocative methods is the uncertainty regarding their effective application to patients and examiners. False negatives have been reported in PFO detection cases where the VM was improperly executed.^14^

**Figure 1:**
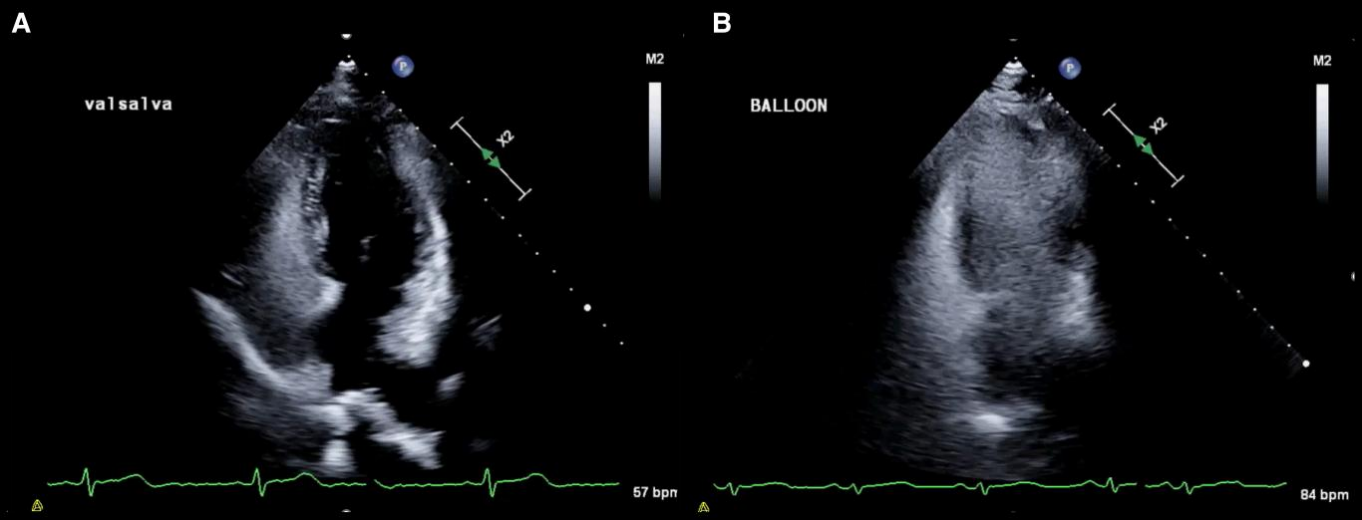
Effective case of the PBIM. The four-chamber view image during ASC-TTE in 58-year-old woman with cryptogenic stroke initially exhibited microbubble Grade 1 using standard provocative manoeuvres (*A*, *Video 1*). Upon performing the PBIM, the microbubble grade increased to Grade 4 (*B*, *Video 2*). The patient subsequently underwent a transcatheter PFO closure procedure. ASC-TTE, agitated saline contrast transthoracic echocardiography; PFO, patent foramen ovale.

Consequently, we developed the party balloon inflation manoeuvre (PBIM) for detecting PFO.^15^ The PBIM is a novel manoeuvre for inflating a party balloon instead of the VM during ASC-TTE examination. It is simple to explain to patients and provides visual feedback to confirm correct manoeuvre execution. In addition, the 30-mm-wide party balloon inflation provides 20–25 mmHg of positive end-expiratory pressure to the patient’s airway.^15^ This inflation effectively increases intrathoracic pressure, decreases venous return, and enhances venous return to the RA during the release or inhalation compared with the conventional VM.^16^ Therefore, the PBIM approach may surpass the conventional VM in detecting RL shunts in patients with PFO (*Figure 1*, *Videos 1* and *2*). The PBIM approach may be beneficial over standard provocative methods and could be a potentially helpful addition to the workflow for busy echo laboratories using a simple tool. However, only a few case reports exist on the PBIM, and evidence of its efficacy in a real-world setting is yet to be established. Therefore, in this multicentre registry in Japan, we aimed to statistically elucidate whether the PBIM provides superior PFO detection during ASC-TTE compared with standard provocative methods, such as the conventional or abdominal compression VM.

**Figure 2:**
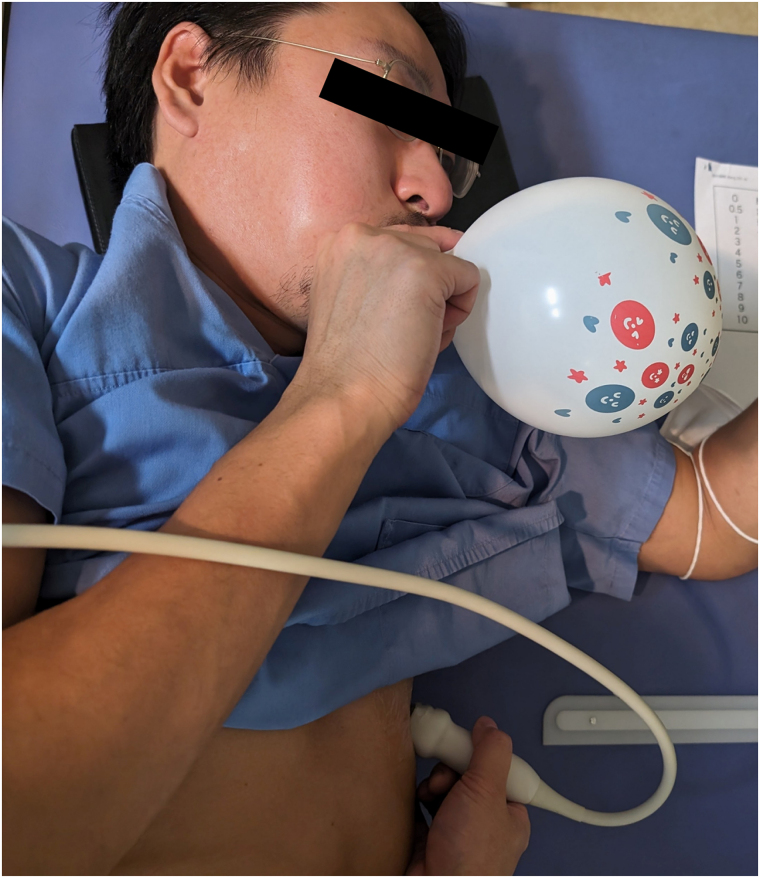
PBIM. The balloon was inflated to a target diameter of > 10 cm, bubbles were injected into the vein simultaneously, and the blow was maintained for > 5 s. The release or inhalation was performed when the bubbles entered the RA.

## Methods

### Study design and patients

The Impact of PBIM during the saLine contrAst Transthoracic Echocardiography for detecting Patent Foramen Ovale (INFLATE-PFO) registry is a multicentre retrospective observational study of PBIM use in ASC-TTE. It is the world’s first mass evidence of using PBIM for PFO detection.^17^ Data were collected from eight leading Japanese centres (Teikyo University, St. Marianna University, Asahi General Hospital, National Cerebral and Cardiovascular Centre, Showa University, Teine Keijinkai Hospital, Chikamori Hospital, and Gunma Prefectural Cardiovascular Centre), which quickly adopted and implemented the PBIM in clinical settings, between June 2021 and December 2023. The INFLATE-PFO registry included 117 consecutive adult patients with TEE-confirmed PFO who underwent the PBIM and standard provocative methods, including the conventional and abdominal compression VM, to demonstrate the RL shunt during ASC-TTE. The pre-determined exclusion criterion was the absence of sufficient information, including missing data for patient demographics, microbubble grades, or echocardiographic parameters required for analysis. No patient was excluded from the final analysis.

### Ethics approval and consent

This study protocol was developed following the 1975 Declaration of Helsinki and its later amendments and was comprehensively approved by the Teikyo University Institutional Review Board (21 June 2023, reference number TEIDAI 23-003). This trial was registered with the University Hospital Medical Information Network (UMIN000051954). The requirement for obtaining patient consent was waived because this study was conducted as part of a registry.

### ASC-TTE and PBIM protocol

**Figure 3:**
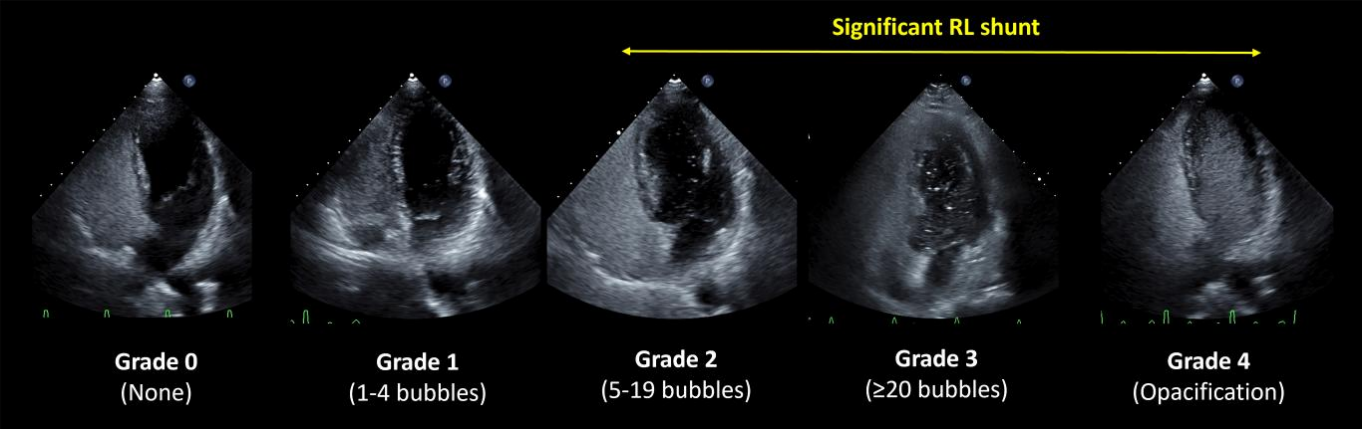
Five-point microbubble grade. RL shunt graded using a five-point microbubble grading using ASC-TTE. The five-point microbubble grade was based on the quantification of the maximum number of microbubbles observed in the left-side heart on one still frame, regardless of the timing in the cardiac cycle, as follows: Grade 0 (none), Grade 1 (<5 bubbles), Grade 2 (6–19 bubbles), Grade 3 (≥20), and Grade 4 (opacification, visualisation of the entire left-side heart chamber). Grade ≥ 2 is considered a significant RL shunt.

ASC was produced in standard proportions (10% air, 10% blood, and 80% saline), which enhanced the contrast appearance.^18^ It was injected into the antecubital vein, and the VM was released immediately upon RA opacification. Notably, some institutes applied the abdominal compression VM when necessary, such as when the conventional VM was insufficient.^12,13^ According to our previous case reports and other studies, the PBIM protocol in this registry involved inflating the balloon to a target diameter of > 10 cm and injecting bubbles into the vein simultaneously (*Figure 2*).^8,15,16,19^ In addition, it was recommended that the blow should be maintained for > 5 s, and the release or inhalation of the PBIM should be performed when the bubbles enter the RA.^20^ Furthermore, it was strongly recommended that patients practice inflating the balloon just before performing the actual bubble test. This protocol was explained and shared with all investigators through web or onsite training. A unified scheme for quantifying RL shunts through a PFO was assessed in five cardiac cycles following the release phase of the provocation. This scheme was proposed and classified into five grades recommended and generically used in Japan based on the quantification of the maximum number of microbubbles observed in the left-side heart using an apical four-chamber view on one still frame, regardless of the timing in the cardiac cycle. The grade quantification is Grade 0, no bubbles; Grade 1, 1–4 bubbles; Grade 2, 5–19 bubbles; Grade 3, ≥ 20 bubbles; and Grade 4, bubble opacification in the four-chamber view. Grade ≥ 2 is a significant RL shunt, indicating transcatheter PFO closure (*Figure 3*).^21,22^ An experienced attending echocardiologist evaluated the grade quantification agreed upon by each institution’s heart team. Standard provocative methods are usually performed before the PBIM, although the sequence of these manoeuvres among facilities is not fixed. In addition, a heart team at each institution assessed the sufficiency of balloon inflation during the PBIM.

### Demographic and other echocardiographic data collection

Clinical data, including age, sex, body surface area, documented diagnosis of hypertension, diabetes mellitus, dyslipidaemia, chronic obstructive pulmonary disease, heart failure, transient ischaemic attack, thromboembolism, and migraine, congestive heart failure (1 point), hypertension (1 point), age >75 years (1 point), diabetes mellitus (1 point), and history of stroke transient ischaemic attack or embolic event (2 points) (CHADS2) scores; and the risk of paradoxical embolism scores, were collected from patient’s medical records.^23,24^ All participants underwent standard two-dimensional B-mode and Doppler transthoracic echocardiography before ASC-TTE. Conventional parameters, such as the left ventricular diastolic and systolic dimension and volume, left ventricular ejection fraction, left atrial volume index, E/e′, and tricuspid regurgitation peak gradient, were measured according to the American Society of Echocardiography guidelines.^25^ Based on the ASC-TTE results, we also investigated whether each patient underwent a transcatheter PFO closure procedure, which was determined by the Brain-Heart Team after considering factors such as age and other clinical parameters.

### Study endpoints

This study’s primary endpoint was to compare the five-point microbubble grade and significant RL shunt between the PBIM and the conventional or abdominal compression VM during ASC-TTE. The higher grade was determined when different grades were obtained for the same patient using the two different standard provocative methods (Graphical abstract). In the sub-groups, the five-point microbubble grade and significant RL shunt were compared between the PBIM and conventional VM, as well as between the PBIM and abdominal compression VM. Microbubble ‘upgrading’ is defined as an elevation in the five-point microbubble grading scale because of implementation, while ‘downgrading’ refers to a reduction. ‘No change’ indicates that the grading remained constant.

### Statistical analyses

Data are presented as means ± standard deviations for continuous variables and numerical values and percentages for categorical variables. The differences between the five microbubble grades in the two provocative methods (PBIM vs. standard provocative methods and conventional VM vs. abdominal compression VM) were analysed using the paired Wilcoxon test. Subsequently, using McNemar’s test, we determined whether the two methods differed significantly regarding the diagnostic performance of a significant RL shunt. The effect of clinical and echocardiographic variables on predicting RL shunt downgrading in the PBIM compared with standard provocative methods was assessed using logistic regression analysis. Age, sex, and clinically relevant echocardiographic variables were incorporated into the multivariate models using a stepwise variable selection method. Variables were entered or removed if *P* < 0.05 or *P* > 0.1, respectively. All statistical analyses were performed using R software (version 4.2.3; R Development Core Team, Vienna, Austria) and MedCalc software ver. 20.2 (MedCalc Software, Ostend, Belgium). Statistical significance was set at *P* < 0.05.

## Results

### Participant characteristics

All patients who underwent the PBIM (*n* = 117) were compared with those who underwent standard provocative methods (*n* = 117; conventional and abdominal compression VMs = 84, abdominal compression VM alone = 23, and conventional VM alone =10) (Graphical abstract). *Table 1* presents the patients’ baseline characteristics and echocardiographic parameters. PFO was detected in all 117 patients using TTE, while an atrial septal aneurysm was confirmed in 32 patients (27.4%). During ASC-TTE, all patients successfully underwent PBIM and standard provocative methods. No adverse events were reported during the PBIM, and a leftward intra-atrial septal motion was observed in 90 (76.9%) patients. However, insufficient balloon inflation was observed in 8 (6.8%) patients.

**Table 1:** Patient characteristics and echocardiographic data (***N* = 117)**

Patient characteristic	Value
Age, years	56.2 ± 13.8
Sex, Male, %	73 (62.3)
Body surface area, m^2^	1.68 ± 0.18
Normal sinus rhythm, %	117 (100)
Transient ischaemic attack, %	7 (6.0)
Thromboembolism, %	104 (88.9)
Migraine, %	9 (7.7)
CHADS2 score (0/1/2/≥3), %	3/4/57/53 (2.6/3.4/48.7/45.3)
RoPE score	5.3 ± 2.0
Transcatheter PFO closure procedure	81 (69.2)
**Echocardiographic data**	
Left ventricular diastolic volume, mL	91.7 ± 30.1
Left ventricular systolic volume, mL	35.5 ± 19.1
Left ventricular ejection fraction, %	62.4 ± 7.9
Left atrial volume index, mL/m^2^	29.2 ± 13.8
E/e′	7.4 ± 2.4
TRPG, mmHg	19.0 ± 4.9
PFO confirmed by TEE, %	117 (100)
Atrial septal aneurysm confirmed by TEE, %	32 (27.4)
Leftward intra-atrial septal motion in PBIM, %	90 (76.9)
Insufficient balloon inflation due to inexperience in PBIM, %	8 (6.8)

Data are expressed as means ± standard deviations or as numbers (percentages).

CHADS2, congestive heart failure, hypertension, age > 75 years, diabetes mellitus, and history of stroke transient ischaemic attack or embolic event; PBIM, party balloon inflation manoeuvre; PFO, patent foramen ovale; RoPE, risk of paradoxical embolism; TEE, transoesophageal echocardiography; TRPG, tricuspid regurgitation peak gradient.

### Comparison of microbubble grades

*Table 2* presents the agreement between the PBIM and standard provocative methods on the five-point microbubble grades of the RL shunt in each cohort. Compared with the standard provocative methods, the PBIM resulted in microbubble upgrading in 51 (43.6%, yellow panels) patients, no change in 54 (46.1%, white panels), and downgrading in 12 (10.3%, blue panels) (Graphical abstract and *Table 2*). Wilcoxon paired analysis revealed that the PBIM had a significantly higher microbubble grade than the standard provocative methods [median, 3.0; interquartile range (3.0–4.0) vs. 3.0; (1.7–4.0); *P* < 0.001].

**Table 2:** Five-point microbubble grades of the RL shunt in PBIM and standard provocative methods

Standard provocative methods (*n* = 117) (Conventional and abdominal compression VMs)	PBIM					
	Grade 0 (None)	Grade 1 (1–4 bubbles)	Grade 2 (5–19 bubbles)	Grade 3 (≥ 20 bubbles)	Grade 4 (Opacification)	Total
**Grade 0 (None)**	5 (4.2%)	1 (0.8%)	6 (5.1%)	2 (1.7%)	2 (1.7%)	16 (13.7%)
**Grade 1 (1–4 bubbles)**	0 (0%)	1 (0.8%)	4 (3.4%)	5 (4.2%)	3 (2.5%)	13 (11.1%)
**Grade 2 (5–19 bubbles)**	1 (0.8%)	1 (0.8%)	6 (5.1%)	6 (5.1%)	3 (2.5%)	17 (14.5%)
**Grade 3 (≥ 20 bubbles)**	0 (0%)	1 (0.8%)	2 (1.7%)	14 (11.9%)	19 (16.2%)	36 (30.8%)
**Grade 4 (Opacification)**	0 (0%)	0 (0%)	0 (0%)	7 (5.9%)	28 (23.9%)	35 (29.9%)
**Total**	6 (5.1%)	4 (3.4%)	18 (15.4%)	34 (29.1%)	55 (47.0%)	117 (100%)

PBIM, party balloon inflation manoeuvre; RL, right-to-left; VM Valsalva manoeuvre.

Yellow panels indicate upgrading, white indicates no change, and blue indicates downgrading.

Regarding the sub-groups, the PBIM resulted in microbubble upgrading in 52 (55.3%) patients, no change in 37 (39.4%), and downgrading in 5 (5.3%), compared with the conventional VM (see Supplementary data online, *Table S1*). Similarly, when comparing the PBIM with the abdominal compression VM, microbubble upgrading was observed in 47 (43.9%) patients, no change in 48 (44.9%), and downgrading in 12 (11.2%) (see Supplementary data online, *Table S2*). The PBIM had a significantly larger microbubble grade than the conventional [median, 3.5 (2.0–4.0) vs. 2.0 (1.0–4.0); *P* < 0.001] and abdominal compression [median, 3.0 (2.2–4.0) vs. 3.0 (1.0–4.0); *P* < 0.001] VMs.

### Diagnostic performance-identifying significant RL shunt

*Table 3* presents the diagnostic performance of a significant RL shunt (Grade ≥ 2) during ASC-TTE in the PBIM and standard provocative methods (as references). The pairwise McNemar’s test showed that the PBIM increased the diagnostic performance of a significant RL shunt from 75.2% achieved using standard methods to 91.5% (improvement rate 16.3%, *P* < 0.001) (Graphical abstract and *Table 3*). In the sub-group analysis, the diagnostic performance of a significant RL shunt increased from 67.0% when using the conventional VM to 89.4% when using the PBIM (improvement rate, 22.4%; *P* < 0.001) (see Supplementary data online, *Table S3*). When compared with the abdominal compression VM, the diagnostic performance of a significant RL shunt increased from 72.9% to 90.7% when using the PBIM (improvement rate, 17.8%; *P* < 0.001) (see Supplementary data online, *Table S4*).

**Table 3:** Diagnostic performance of a significant RL shunt between PBIM and standard provocative methods

	PBIM		
Standard provocative methods (*n* = 117) (Conventional and abdominal compression VMs)	Significant RL shunt (Grades 2–4)	Non-significant RL shunt (Grade 0 or 1)	Total
**Significant RL shunt (Grades 2–4)**	85 (72.6%)	3 (2.5%)	88 (75.2%)
**Non-significant RL shunt (Grade 0 or 1)**	22 (18.8%)	7 (5.9%)	29 (24.8%)
**Total**	107 (91.5%)	10 (8.5%)	117 (100%)

Diagnostic performance was defined by the identification of significant RL shunt (Grades 2–4) using standard provocative methods as reference. PBIM, party balloon inflation manoeuvre; RL, right-to-left; VM, Valsalva manoeuvre.

### Comparison between the conventional and abdominal compression VMs

Of the 84 cases in which the conventional and abdominal compression VMs were performed, the abdominal compression VM produced a significantly larger microbubble grade than the conventional VM [median, 3.0 (1.0–4.0) vs. 2.0 (1.0–3.5), *P* < 0.01] (see Supplementary data online, *Table S5*). Compared with the conventional VM, the abdominal compression VM produced microbubble upgrading, no change, and downgrading in 22 (26.2%), 59 (70.2%), and 3 (3.6%) patients, respectively. Furthermore, the diagnostic performance of the conventional VM in identifying a significant RL shunt (65.5%) did not differ from that of the abdominal compression VM (72.6%) (*P* = 0.109) (see Supplementary data online, *Table S6*).

### Predictors of downgrading by the PBIM

*Table 4* presents the predictors of the downgrading of the microbubble grade by the PBIM. Logistic regression analysis showed that insufficient balloon inflation was strongly associated with the downgrading of the microbubble grade by the PBIM after adjusting for age, sex, clinically relevant variables, and other echocardiographic parameters (odds ratio, 24.0; 95% confidence interval, 3.34–172.1; *P* < 0.01).

**Table 4:** Predictors of PBIM downgrading

	Univariate analysis			Multivariate analysis		
Variable	OR	95% CI	*P*-value	OR	95% CI	*P*-value
*Clinical data*						
Age (per 10-year increase)	1.10	0.65–1.86	0.713			
Sex, Female	1.43	0.40–5.00	0.573			
Body surface area (per 0.1 m^2^ increase)	1.06	0.75–1.50	0.717			
CHADS2 score (per 1 score increase)	1.44	0.77–2.70	0.247			
*Echocardiographic data*						
Left ventricular ejection fraction (per 1% increase)	1.04	0.95–1.15	0.356			
E/e′ (per 1 increase)	1.12	0.88–1.41	0.343			
Insufficient balloon inflation	7.57	1.52–37.6	0.013	24.0	3.34–172.1	0.002
Leftward intra-atrial septal motion	1.28	0.31–5.20	0.729			

CHADS2, congestive heart failure, hypertension, age > 75 years, diabetes mellitus, and history of stroke transient ischaemic attack or embolic event; CI, confidence interval; OR, odds ratio; PBIM, party balloon inflation manoeuvre.

### Transcatheter PFO closure procedure

Of the 117 enrolled cases, 81 patients (69.2%) underwent transcatheter PFO closure procedures (*Table 1*). Of the 22 patients who initially had a non-significant RL shunt with standard provocative manoeuvres but demonstrated a significant RL shunt with PBIM, 12 (54.5%) proceeded with transcatheter PFO closure.

## Discussion

This study provides the first mass evidence supporting the PBIM use in ASC-TTE for detecting PFO. First, no adverse events were reported during the PBIM, indicating its safety. Second, in ASC-TTE assessments aimed at evaluating RL shunts in patients with PFO, the PBIM showed significantly higher microbubble grades than the established conventional and abdominal compression VMs. Moreover, as 45.5% (10 of 22 cases) shifted to ‘significant’ at only Grade 2, it markedly improved the detection rate of significant RL shunts. The abdominal compression VM is considered more effective than the VM alone, and it yielded significantly higher grades than the VM alone. However, the proportion of patients who had microbubble upgrading was low, and substantial improvement in the detection capability of significant RL shunts was not observed. In addition, the main reason for the decrease in the bubble grade with the PBIM was insufficient balloon inflation due to patient factors.

The primary characteristic of the PBIM lies in its use of an inexpensive and straightforward tool known as a party balloon, which ensures a reliable VM that is discernible to patients and examiners. Our previous findings indicated that a 30-cm balloon achieves a pressure equivalent to the positive end-expiratory pressure (20–25 mmHg) where inflation (exhalation) is released. This leads to negative pressure in the thoracic cavity during subsequent inhalations, thereby increasing venous return to the RA.^15,16,26^ The assured provocative loading likely contributed to a significant increase in the microbubble grade compared with standard provocative methods.

In addition, the detection rate of significant RL shunts improved by 16%. This indicates that 16% of patients might have been underestimated using the conventional standard provocation methods. Furthermore, without this additional detection, these patients might have been deemed unsuitable for transcatheter PFO closure in clinical practice, potentially depriving them of appropriate treatment. In our investigation, 10 of the 22 patients who demonstrated improved detection with PBIM were deemed ineligible for transcatheter PFO closure by the Brain-Heart Team due to factors such as age (eligibility is typically considered optimal for patients aged ≤ 60 years in Japan), or they were eligible but opted not to undergo the procedure. However, 12 patients (54.5%) who would have previously been considered ineligible were able to undergo transcatheter PFO closure and likely benefited from the treatment. Therefore, this simple PBIM technique is strongly recommended as an essential adjunctive method for patients previously classified as Grade < 2 non-significant RL shunts using conventional standard provocative methods.

### Other advantages of the PBIM

The PBIM used across the participating facilities presents several notable advantages. First, it eliminates the need for personnel to administer abdominal compression during the examination. Thus, reducing the required human resources compared with the abdominal compression VM. Second, its visual demonstration makes it more accessible to patients with hearing impairments, older adults, or individuals with a limited understanding of the VM due to language barriers. Third, it proves beneficial for patients who may struggle with VM, such as those who have experienced a stroke or wear dentures. The PBIM can also be used in scenarios where abdominal compression is contraindicated, such as in cases involving abdominal pain, the presence of percutaneous tubes, or after recent abdominal surgery.^8,12,13^

Furthermore, compared with the standard provocative methods, the PBIM, which involves repetitive balloon inflation and inhalation for further expansion, allows multiple considerations of the ASC injection timing within a single event. Therefore, PBIM-based ASC-TTE examinations provide a practical approach in the echo laboratory due to their numerous advantages.

### Factors contributing to PBIM downgrading

Despite the high effectiveness of the PBIM, a decrease in microbubble grading was observed in 12 cases compared with the standard provocative methods. This downgrade was attributed primarily to insufficient balloon inflation due to patient factors, such as inexperience observed in eight patients. These eight patients, as well as the 12 with downgrading, may not achieve a constant 20–25 mmHg positive end-expiratory pressure. This factor underscores the importance of ensuring adequate balloon loading during the procedure, indicating its effectiveness since many individuals may not have frequent opportunities to inflate balloons. Therefore, practising balloon inflation before bubble testing is crucial to ensure the reproducibility and stability of the PBIM.

### PFO diction by TEE

TEE remains the gold standard for the diagnosis of PFO, and it is still indispensable for detailed anatomical assessment, particularly in the context of planning catheter-based interventions.^27^ However, during contrast studies, TEE may reduce the success rate of RL shunt detection due to impaired VM caused by probe insertion.^8–11^ Moreover, PBIM cannot be performed under probe insertion, and therefore abdominal or inferior vena cava compression is generally recommended to enhance shunt detection during TEE.^12,13^ This study focused solely on ASC-TTE, and did not include a comparative evaluation with ASC-TEE. This represents one of the limitations of our study.

### Another VM technique

Kumar *et al*.^28^ utilized a syringe modified with a manometer at the tip to reliably achieve a 40-mmHg pressure for 10 s to perform a VM to unmask left ventricular outflow tract obstruction during Valsalva in patients with hypertrophic cardiomyopathy. This method, termed ‘Goal-Directed Valsalva’, is a standardized way to reduce preload.^28^ This method is a much more controlled provocative manoeuvre than the PBIM and may also be applicable in ASC-TTE for detecting PFO. However, since the PBIM uses inexpensive and simple tools, it can be adopted by many echo laboratories. Therefore, in Japan, the PBIM is widely recognized and performed in many institutions. However, a quantifiable measure of adequate inflation, along with practical troubleshooting techniques, could enhance reproducibility across diverse clinical settings. To address this, we are currently conducting *in vitro* and *in vivo* studies to quantify the PBIM method, and we plan to publish the results upon completion in the near future.

### Limitations

This study had some limitations and potential biases due to its retrospective observational nature. First, the study’s design focused on comparing bubble grades between standard provocative methods and the PBIM in patients with suspected paradoxical embolic stroke and confirmed PFO. Therefore, patients with unconfirmed PFO were not included in this study, rendering the PFO detection rate unclear. Furthermore, as only patients who successfully underwent both PBIM and standard provocative methods were retrospectively included, there is a potential for selection bias. Second, as this was an exploratory multicentre retrospective observational registry study, we did not perform a formal *a priori* power calculation to determine the sample size. However, a *post hoc* analysis based on the observed discordant pairs in McNemar’s test indicated that our sample size of 117 patients achieved over 99% power to detect the observed effect size (Cohen’s *w* = 3.8, *P* < 0.001). Third, the sequence of applying standard provocative methods and PBIM was not standardized across participating centres; in most cases, PBIM was performed after the standard provocative method. This fixed order may have introduced order bias, potentially favouring PBIM by allowing improved performance on the second attempt or by residual contrast effects. Therefore, the observed superiority of PBIM should be interpreted with caution in light of this possibility. Fourth, there was no centralized core laboratory analysis for shunt grading. Because this study was a retrospective observational registry, the assessment of RL shunt presence and grade was performed independently at each participating institution. Although a five-point grading system recommended by the Japanese Circulation Society was used, and an experienced attending echocardiologist at each centre evaluated the findings in agreement with the institutional heart team, inter-institutional variability cannot be fully excluded. Fifth, the PBIM is actively used in the Japanese clinical setting to detect PFO through transcranial Doppler ultrasonography. However, this aspect was not investigated in the ASC-TTE registry used in this study. Evidence-building is essential in transcranial Doppler ultrasonography to further demonstrate the efficacy of the PBIM. Finally, our results, derived from the data collected in Japan, may not be generalisable to other geographical regions. Consequently, to address these limitations, future research endeavours should encompass a broader spectrum, including patients without PFO, comparative analyses between ASC-TTE and ASC-TEE, evaluations of the PBIM with transcranial Doppler ultrasonography, and multicentre studies with multiethnicity cohort aimed at wider applicability, along with assessments of clinical outcomes over time.

## Conclusions

The PBIM significantly increased the microbubble grade compared with standard provocative methods. It represents a promising treatment strategy for patients with potential cryptogenic strokes attributed to PFO, particularly those who were falsely diagnosed as negative for significant RL shunt and did not receive adequate treatment. This method demonstrates greater efficacy than standard provocative methods and provides practicality within the workflow of busy echo laboratories using a straightforward tool.

## Supplementary Material

qyaf080_Supplementary_Data

## Data Availability

The data, analytic methods, and study materials will be made available to other researchers for the purposes of reproducing these results or replicating these procedures from the corresponding author upon reasonable request.

## Lead author biography

Dr Akihisa Kataoka graduated from Kochi Medical School, Japan. He is currently an Associate Professor in the Department of Medicine, Division of Cardiology, at Teikyo University in Tokyo, Japan. His clinical expertise lies in echocardiographic imaging, with a particular focus on structural heart disease. He has also successfully developed several clinically useful tools, including the Party Balloon Inflation Manoeuvre used in this study, a hand model for anatomical education, and a specialized radiation protection shield for interventional echocardiographers.
