# Supplementary material for: Impact of party balloon inflation manoeuvre during saline contrast transthoracic echocardiography for patent foramen ovale detection: INFLATE-PFO registry
Source: Eur Heart J Imaging Methods Pract. 2025 Jun 12;3(1):qyaf080. doi: 10.1093/ehjimp/qyaf080 (PMC12378599; doi:10.1093/ehjimp/qyaf080)
Supplement: qyaf080_Supplementary_Data [file qyaf080_Supplementary_Data.docx]

**Supplementary Data**

**Table S1. Five-point microbubble grades of the RL shunt in PBIM and conventional VM**

| **Conventional VM (n=94)** | **PBIM** | | | | | |
| --- | --- | --- | --- | --- | --- | --- |
|  | **Grade 0**  **(None)** | **Grade 1**  **(1–4 bubbles)** | **Grade 2**  **(5–19 bubbles)** | **Grade 3**  **(≥ 20 bubbles)** | **Grade 4**  **(Opacification)** | **Total** |
| **Grade 0 (None)** | 5 (5.3%) | 1 (1.0%) | 6 (6.3%) | 3 (3.2%) | 2 (2.1%) | 17 (18.1%) |
| **Grade 1 (1–4 bubbles)** | 0 (0%) | 2 (2.1%) | 6 (6.3%) | 4 (4.2%) | 2 (2.1%) | 14 (14.9%) |
| **Grade 2 (5–19 bubbles)** | 1 (1.0%) | 1 (1.0%) | 3 (3.2%) | 7 (7.4%) | 8 (8.5%) | 20 (21.3%) |
| **Grade 3 (≥ 20 bubbles)** | 0 (0%) | 0 (0%) | 0 (0%) | 5 (5.3%) | 13 (13.8%) | 18 (19.1%) |
| **Grade 4 (Opacification)** | 0 (0%) | 0 (0%) | 0 (0%) | 3 (3.2%) | 22 (23.4%) | 25 (26.6%) |
| **Total** | 6 (6.4%) | 4 (4.3%) | 15 (16.0%) | 22 (23.4%) | 47 (50.0%) | 94 (100%) |

PBIM, party balloon inflation manoeuvre; RL, right-to-left; VM, Valsalva manoeuvre

The yellow panels indicate upgrading, white indicates no change, and blue indicates downgrading.

**Table S2. Five-point microbubble grades of the RL shunt in PBIM and abdominal compression VM**

| **Abdominal compression VM (n=107)** | **PBIM** | | | | | |
| --- | --- | --- | --- | --- | --- | --- |
|  | **Grade 0**  **(None)** | **Grade 1**  **(1–4 bubbles)** | **Grade 2**  **(5–19 bubbles)** | **Grade 3**  **(≥ 20 bubbles)** | **Grade 4**  **(Opacification)** | **Total** |
| **Grade 0 (None)** | 5 (4.6%) | 1 (0.9%) | 6 (5.6%) | 2 (1.8%) | 4 (3.7%) | 18 (16.8%) |
| **Grade 1 (1–4 bubbles)** | 0 (0%) | 1 (0.9%) | 3 (2.8%) | 5 (4.6%) | 2 (1.8%) | 11 (10.3%) |
| **Grade 2 (5–19 bubbles)** | 1 (0.9%) | 1 (0.9%) | 6 (5.6%) | 6 (5.6%) | 2 (1.8%) | 16 (15.0%) |
| **Grade 3 (≥ 20 bubbles)** | 0 (0%) | 1 (0.9%) | 2 (1.8%) | 14 (13.0%) | 16 (16.2%) | 33 (30.8%) |
| **Grade 4 (Opacification)** | 0 (0%) | 0 (0%) | 0 (0%) | 7 (6.5%) | 22 (20.5%) | 29 (27.1%) |
| **Total** | 6 (5.6%) | 4 (3.7%) | 17 (15.9%) | 34 (31.8%) | 46 (43.0%) | 107 (100%) |

PBIM, party balloon inflation manoeuvre; RL, right-to-left; VM Valsalva manoeuvre

The yellow panels indicate upgrading, white indicates no change, and blue indicates downgrading.

**Table S3. Diagnostic performance of a significant RL shunt between PBIM and conventional VM**

|  | **PBIM** | | |
| --- | --- | --- | --- |
| **Conventional VM (n=94)** | **Significant RL shunt**  **(Grades 2–4)** | **Non-significant RL shunt (Grade 0 or 1)** | **Total** |
| **Significant RL shunt (Grades 2–4)** | 61 (64.9%) | 2 (2.1%) | 63 (67.0%) |
| **Non-significant RL shunt (Grade 0 or 1)** | 23 (24.5%) | 8 (8.5%) | 31 (33.8%) |
| **Total** | 84 (89.4%) | 10 (10.6%) | 94 (100%) |

Diagnostic performance was defined by the identification of significant RL shunt (Grades 2–4) using standard provocative methods as reference. PBIM, party balloon inflation manoeuvre; RL, right-to-left; VM, Valsalva manoeuvre

**Table S4. Diagnostic performance of a significant RL shunt between PBIM and abdominal compression VM**

|  | **PBIM** | | |
| --- | --- | --- | --- |
| **Abdominal compression VM (n=107)** | **Significant RL shunt**  **(Grades 2–4)** | **Non-significant RL shunt (Grade 0 or 1)** | **Total** |
| **Significant RL shunt (Grades 2–4)** | 75 (70.1%) | 3 (2.8%) | 88 (72.9%) |
| **Non-significant RL shunt (Grade 0 or 1)** | 22 (20.6%) | 7 (6.5%) | 29 (27.1%) |
| **Total** | 97 (90.7%) | 10 (9.3%) | 107 (100%) |

Diagnostic performance was defined by the identification of significant RL shunt (Grades 2–4) using standard provocative methods as reference. PBIM, party balloon inflation manoeuvre; RL, right-to-left; VM, Valsalva manoeuvre

**Table S5. Five-point microbubble grades of the RL shunt between the abdominal compression and conventional VMs**

| **Conventional VM** | **Abdominal compression VM** | | | | | |
| --- | --- | --- | --- | --- | --- | --- |
|  | **Grade 0**  **(None)** | **Grade 1**  **(1–4 bubbles)** | **Grade 2**  **(5–19 bubbles)** | **Grade 3**  **(≥20 bubbles)** | **Grade 4**  **(Opacification)** | **Total** |
| **Grade 0 (None)** | 13 (15.4%) | 2 (2.4%) | 0 (0%) | 1 (1.2%) | 1 (1.2%) | 17 (20.2%) |
| **Grade 1 (1–4 bubbles)** | 0 (0%) | 6 (7.1%) | 6 (7.1%) | 0 (0%) | 0 (0%) | 12 (14.3%) |
| **Grade 2 (5–19 bubbles)** | 0 (0%) | 0 (0%) | 7 (8.3%) | 12 (14.3%) | 0 (0%) | 19 (22.6%) |
| **Grade 3 (≥20 bubbles)** | 1 (1.2%) | 0 (0%) | 0 (0%) | 12 (14.3%) | 2 (2.4%) | 15 (17.9%) |
| **Grade 4 (Opacification)** | 1 (1.2%) | 0 (0%) | 0 (0%) | 1 (1.2%) | 19 (22.6%) | 21 (25.0%) |
| **Total** | 15 (17.9%) | 8 (9.5%) | 13 (15.5%) | 26 (31.0%) | 22 (26.2%) | 84 (100%) |

RL, right-to-left; VM, Valsalva manoeuvre

**Table S6. Diagnostic performance of a significant RL shunt between the abdominal compression and conventional VMs**

|  | **Abdominal compression VM** | | |
| --- | --- | --- | --- |
| **Conventional VM** | **Significant RL shunt**  **(Grades 2–4)** | **Non-significant RL shunt (Grade 0 or 1)** | **Total** |
| **Significant RL shunt (Grades 2–4)** | 53 (63.1%) | 2 (2.4%) | 55 (65.5%) |
| **Non-significant RL shunt (Grade 0 or 1)** | 8 (9.5%) | 21 (25.0%) | 34 (24.5%) |
| **Total** | 61 (72.6%) | 23 (27.4%) | 84 (100%) |

Diagnostic performance was defined by the identification of significant RL shunt (Grade 2–4) using conventional VM as reference. RL, right-to-left; VM Valsalva manoeuvre
